# A Cross-Sectional Cohort Study of a Large, Statewide Medicaid Home and Community-Based Services Autism Waiver Program

**DOI:** 10.1007/s10803-014-2217-4

**Published:** 2014-09-03

**Authors:** Karen Goldrich Eskow, Gregory S. Chasson, Jean Ann Summers

**Affiliations:** 1 Department of Family Studies and Community Development, Towson University, 8000 York Road, Towson, MD 21252 USA; 2 Department of Psychology, Towson University, Towson, MD USA; 3 Schiefelbusch Institute for Life Span Studies, University of Kansas, Lawrence, KS USA

**Keywords:** Autism services, Medicaid, Home and community-based services waiver, Family quality of life

## Abstract

State-specific 1915(c) Medicaid Home and Community-Based Services waiver programs have become central in the provision of services specifically tailored to children with autism spectrum disorders (ASD). Using propensity score matching, 130 families receiving waiver services for a child with ASD were matched with and compared to 130 families waiting on the registry (i.e., control group). Results indicate that participants in the waiver group reported more improvement in independent living skills and family quality of life over the last year compared to those on the registry. More frequent intensive individual support services and therapeutic integration were statistically predictive of improvement in a variety of domains. The results suggest that the waiver program may be promising for improving child and family functioning.

## Introduction

Autism Spectrum Disorders (ASD) are neurological developmental conditions characterized by marked impairment in social interactions, deficits in communications skills, and restricted and repetitive behaviors and interests. These impairments may have a substantial and long-lasting impact on the child, as symptoms of ASD often result in problematic peer interactions, disciplinary infractions, difficulties with school adjustment, and poor academic performance (e.g., Gresham et al. 2006). In addition to the negative impact of ASD on the child, the condition has also been associated with major stressors on the family unit, often more so than among families with children with other developmental disabilities (e.g., Down syndrome; Brown et al. 2006). Research has consistently demonstrated a link between child behavior problems and decreased family well-being (e.g., Eisenhower and Blacher 2006; Turnbull et al. 2007), as well as between ASD and decreased family productivity (e.g., parental employment; Kogan et al. 2008; Montes and Halterman 2008).

The rise in documented prevalence of ASD over the last couple of decades has dramatically increased the financial burden of caring for individuals with ASD. One of the most costly services for ASD involves residential placement in which the child lives away from the home and receives 24-h care. However, this resource-intensive approach is financially infeasible for most families, as well as for government budgets when spread across a population of children. In an attempt to meet the public demand for more cost efficient services, states in the US have emphasized the central role of the Medicaid program in providing life-long services and supports specifically tailored to individuals with ASD (Mauch et al. 2011). These specialized services are often administered through 1915(c) Medicaid Home and Community-Based Services waiver programs, and there has been increased federal and state interest in expanding these programs to meet the needs of various populations with developmental disabilities, including ASD. State interest is understandable; in 2009, for example, the per person annual cost to Medicaid of serving a person with a disability in an institution was more than four times the cost of serving an individual in the community through a Medicaid Waiver (Lakin et al. 2010; National Council on Disability, n.d.). In a recent report (Eiken and Lelchook 2013), approximately $20.2 billion was spent in the US in 2010 for services tailored to individuals with developmental disabilities, including ASD. Some waiver programs are specific to ASD and not other clinical populations. In 2010, $65 million was spent on these specific ASD waiver programs in the US.

Specifically for ASD, the Maryland waiver program (henceforth referred to as the “Maryland waiver”) is the largest of its kind and includes a set of services identified to meet the needs of a child with ASD and the family unit: (a) service coordination through the local school system; (b) intensive individual support services (i.e., intensive, one-on-one interventions with the child/youth provided by a direct care worker); (c) therapeutic integration (i.e., structured programs focused on expressive therapy and therapeutic recreation provided in a non-residential setting separate from the home); (d) residential habilitation; (e) respite care; (f) environmental accessibility adaptations for the participants’ home (physical changes to the home which are reasonable and medically necessary to assure a safe therapeutic environment, e.g., installing a locked gate in the backyard), (g) family training; and (h) adult life planning services for transition from waiver services to the adult services delivery system. Established in 2001, the Maryland waiver’s slots were filled by 2002. As a result, the state created an autism waiver registry for families that were interested in applying to receive services but were required to wait until a space on the waiver became available and they were identified as eligible to receive services (this group is henceforth referred to as the “registry”). According to the Maryland State Department of Education (MSDE), which implements the Maryland waiver, over 3,400 children were on the registry at the time of the current data collection.

Despite the appeal of waiver services, it remains unclear if this funding is yielding tangible results, as research evidence is limited. In fact, to our knowledge, only two studies—Eskow et al. (2011) and Warfield et al. (2013)—have collected data and published on outcomes of families participating in ASD-specific waiver services. Eskow et al. (2011) compared a group of families receiving Maryland waiver services (*n* = 229) to a group of families on the registry (*n* = 627). The study demonstrated that Maryland’s ASD-specific 1915(c) Medicaid waiver program was statistically associated with improved family quality of life and parental employment status, suggesting some tangible benefits. Using a sample of families receiving services via the Massachusetts autism waiver, Warfield et al. (2013) found that strong collaborative partnerships between families and waiver service providers and officials was related to increased family well-being. These studies were an important first step in establishing an evidence base for a costly and complex service delivery model, but further research is needed for several reasons. First, the Eskow et al. (2011) and Warfield et al. (2013) studies only examined a small subset of outcome variables (e.g., family quality of life and employment status), but waiver services often address ASD impairments across a variety of domains (e.g., academic performance, independent living skills), all of which have remained unstudied. Second, there has been limited research evaluating statistical predictors of improvement^1^ among families who are receiving waiver services. Third, the Warfield et al. (2013) study did not have a control group, and in Eskow et al. (2011), the waiver and registry (i.e., control) groups were unmatched, which could be problematic for ruling out critical lurking variables. Indeed, there are no requirements for being placed on the registry, meaning there is no assurance that registry families have a child with ASD. That is, there is no diagnostic screening for ASD until the child is deemed ready to be placed on the waiver. In addition, some families pay out-of-pocket for waiver-like services (e.g., one-to-one intensive behavior therapy) while waiting on the registry. Thus, to improve the quality of the group comparisons, the use of statistical group-matching procedures would facilitate the drawing of causal inferences based on non-experimental data with a higher level of confidence.

To this end, the current investigation collected data on another sample of Maryland families who were enrolled in the waiver program or were on the registry and waiting for enrollment. As a replication of Eskow et al. (2011) and Warfield et al. (2013), our first hypothesis was that waiver status would be associated with more improvement in family quality of life over the last year compared to registry status. For the second hypothesis, we predicted that waiver status would be associated with increased child improvement over the previous year in academic performance, independent living skills, communication skills, peer relationships, and problematic behavior. For the third hypothesis, in the waiver group, we predicted that more improvement over the last year would be associated with increased frequency or use of environmental accessibility adaptations, intensive individual support services, family training, therapeutic integration services, and respite care.

The current study enhanced the Eskow et al. (2011) methodology in several ways. The current study statistically paired families in the waiver and registry groups using propensity score matching. In addition, missing data and inflated error rates were handled using gold-standard techniques (i.e., multiple imputation and false discovery rate correction, respectively). Thus, the current study aims to establish additional and methodologically stronger evidence for the usefulness of the Maryland ASD waiver program, which, in a sense, is an understudied population-level experiment, with the well-being of children with ASD and their families at stake. Evidence for the effectiveness of the Maryland waiver could provide support for this level and type of fiscal support for ASD services.

## Method

### Participants

**Fig. 1:**
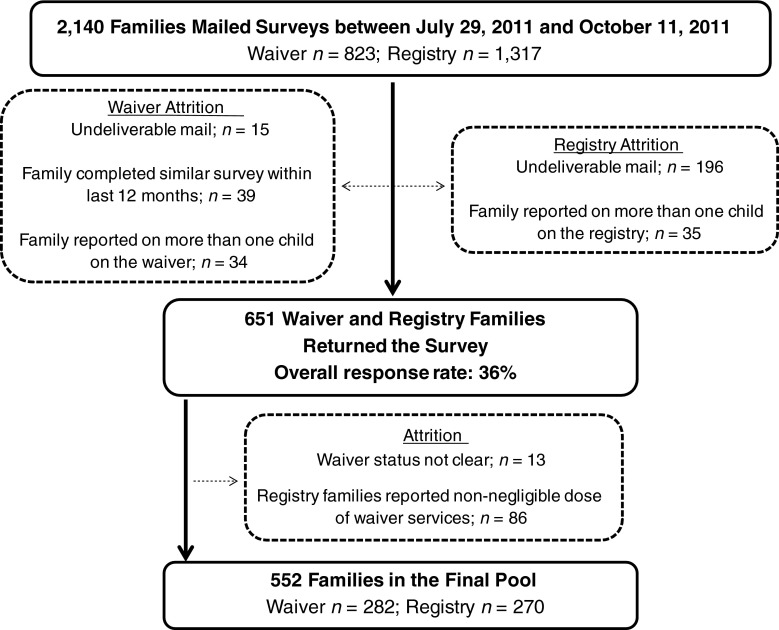
CONSORT chart of survey response rates and data cleaning

A diagram of the survey response rates and reasons for participant exclusion is illustrated in Fig. 1. The registry families were pared down such that children had to be receiving a relatively negligible dose of waiver-like services (i.e., acquired outside of the waiver), thereby framing the registry group as a minimal-services comparison group. Thus, 86 registry families were removed from the sample if they were receiving any family training, or if the child was receiving therapeutic integration services or intensive individual services, such as intensive behavior therapy.

There was a final pool of participants for matching of 552 families (waiver *n* = 282 and registry *n* = 270). We carried out 1:1 propensity score matching to pair families in the waiver group with families in the registry group. Applied with non-experimental data, propensity score matching is a widely used statistical technique for reducing bias when drawing causal inferences about treatment effects when group randomization is not possible or easily obtained (Rosenbaum and Rubin 1983). We matched groups on family household income, child age, the number of years the child has received waivers services or been waiting on the registry, ASD severity, and child sex.

Sample characteristics of the matched groups are provided in Table 1. Children with ASD in the matched waiver group were predominantly male (85 %), and this was also true for the matched registry group (85 %). Family participants were ethnically diverse. Among matched waiver families, 25 % were entirely Caucasian, 40 % had at least one African American family member, 16 % indicated at least one Asian or Pacific Islander family member, 6 % of the households had at least one Latino family member, and 4 % reported at least one American Indian, Eskimo, or Aleut family member. Registry families were similarly diverse, as 14 % were Caucasian, 41 % reported having at least one African American family member, 14 % indicated having at least one Asian or Pacific Islander family member, 6 % of the households had at least one Latino family member, and 2 % reported at least one American Indian, Eskimo, or Aleut family member. The propensity score matching resulted in *n* = 130 for each group. These ethnicity percentages do not add to 100 % for multiple reasons, namely that some categories are not mutually exclusive (e.g., Caucasian and Latino), some survey respondents did not report on ethnicity, and ethnicity data were collected on family composition and not individual participants. Among matched registry families, 84.6 % of the survey respondents were female parental guardians, such as mothers. Similarly, in the matched waiver group, 84.6 % of the survey respondents were female parental guardians, such as mothers.

**Table 1:** Sample characteristics by wavier status and post hoc tests of covariates and dependent variables between the waiver and registry groups

	Total waiver *M*(*SD*)^a^	Matched waiver *M*(*SD*)^b^	Matched registry *M*(*SD*)^b^	*t*	*df*	*d*
Control and match variables						
Child age (years)	15.06 (3.21)	13.97 (3.31)	13.16 (3.56)			
ASD severity	17.22 (2.51)	16.66 (2.92)	16.88 (2.31)			
Years on waiver or registry	5.78 (3.21)	4.91 (3.16)	4.82 (2.65)			
Family income (thousands $)	88.40 (49.40)	79.40 (52.00)	80.00 (45.20)			
Dependent variables						
Academic performance	0.52 (0.50)	0.52 (0.50)	0.41 (0.50)	−1.40	276.44	0.22
Independent living skills	0.54 (0.50)	0.59 (0.50)	0.37 (0.49)	−2.40*	271.59	0.44
Communication skills	0.45 (0.50)	0.47 (0.50)	0.45 (0.25)	0.60	273.25	0.05
Peer relationships	0.25 (0.43)	0.26 (0.44)	0.18 (0.39)	−0.77	274.61	0.19
Problematic behavior	0.50 (0.50)	0.50 (0.25)	0.41 (0.50)	−0.87	268.39	0.23
Family quality of life	4.12 (0.55)	4.02 (0.61)	3.71 (0.63)	−5.08*	271.34	0.50

All inferential statistical tests were carried out with multiple imputation to address missing data; the *t*-statistics and degrees of freedom are pooled based on conventional multiple imputation procedures (Enders, 2010); all degrees of freedom were adjusted based on recommendations and procedures outlined in Barnard and Rubin (1999) and Enders (2010); Type I error rate has been controlled across all inference tests (omnibus, multivariate main effects, and post hoc tests) in the primary analysis using False Discovery Rate correction procedures, resulting in a critical alpha of .029; ASD = autism spectrum disorder; *d* = Cohen’s (1988) standardized group difference effect size. The waiver services “Environmental Accessibility Adaptations” and “Intensive Individual Support Services” were not included in the table because the variables were dichotomized for analysis

^a^ *n* = 282; ^b^ *n* = 130

* *p* < .029

### Procedures

The study took place from June 2011 through May 2012. In collaboration with the Maryland State Department of Education, mailings for the survey were distributed between July 29, 2011 and October 11, 2011 to all Maryland waiver families and a sample of families on the registry. Three separate mailings were made to follow up with non-response, and participants had options to respond via internet survey, paper survey, or phone interview. Families were permitted to respond to the survey only one time. Families were informed that participation was entirely voluntary and their identities would remain anonymous. A random drawing for three $50 gift cards was used as an incentive for participation. The study was approved by the internal review board at the institution of the first author.

### Measures

*Maryland Autism Services Survey (MASS*-*R)*—*Revised*. The study survey consisted of the MASS-R, which is an updated version of the MASS used for studying the Maryland waiver in Eskow et al. (2011). The MASS-R was developed in collaboration with the Maryland State Department of Education, which administers the Maryland waiver, and the Beach Center on Disability at University of Kansas. Feedback from potential participants was elicited from a focus group session with professionals and parents of children with ASD in 2008. The MASS-R is designed to be completed by a parent, guardian, or other parental figure (e.g., grandparent).

The MASS-R is an extensive survey with myriad responses, only a subset of which was applicable for the current study. For the current investigation, MASS-R collected data on waiver status, sex, age of the child with ASD measured in years, family income, and years that the child with ASD has received waiver services or been waiting on the registry. Severity of the child’s ASD was also measured using five Likert-based items: difficulty with academic performance, independent living skills, communication skills, relationships with peers, and problematic behavior. A total score, which ranged from 0 to 20 (higher scores reflect more severity), was derived by summing the five items, and the internal reliability of the total ASD severity index was supported by Cronbach’s α = .783.

Perceived child improvement over the last year was measured in the same five domains as the ASD severity index (i.e., academic performance, independent living skills, communication skills, relationships with peers, and problematic behavior). Ratings of improvement were based on a three-level Likert-based scale. Embedded as a section in the MASS-R, the Family Quality of Life Scale (FQoL) is a 25-item Likert-scale instrument that provides a total score plus five subscales: Family Interaction, Parenting, Emotional Well-Being, Physical/Material Well-being, and Disability-Related Support. The FQoL has demonstrated evidence of adequate reliability (i.e., total score Cronbach’s α = .88, subscale α’s = .74–.90), as well as strong construct validity (Poston et al. 2003; Hoffman et al. 2006). The family’s frequency of utilizing waiver services (i.e., environmental accessibility adaptations, intensive individual support services, therapeutic integration, family training, and respite care) was also measured on a Likert-based scale with six intervals ranging from “not at all” to “daily” use, with higher scores reflecting more frequency of use. Non-waiver services (e.g., special education, physical therapy) were measured using the same type of item structure.

### Data Analyses

Statistical analyses were carried out to evaluate the effectiveness of Maryland waiver services compared to minimal services while waiting on a registry (i.e., control group). Matching variables were also entered as control variables in subsequent omnibus tests, a gold-standard technique that provides “double robustness” (Stuart 2010, p. 13) and has empirical support (e.g., Abadie and Imbens 2006; Rubin 1973, 1979; Stuart 2010). Inflated error rates were addressed by applying a False Discover Rate (FDR) control procedure for determining statistical significance (Benjamini and Hochberg 1995; Verhoeven et al. 2005). Based on this procedure, α_critical_ = .029.

We carried out additional exploratory regression analyses to predict child improvement outcomes from the frequency of different types of waiver services that were received by the family over the last year. The regression models were adjusted by including the following covariates: family household income, child age, ASD severity, child sex and years on the waiver. Distributions of the waiver service frequencies were satisfactory except for environmental accessibility adaptations and intensive individual support services. Both of these indices demonstrated a distribution of responses that suggested a dichotomous indicator would be more appropriate. As such, each was collapsed into a binary frequency variable. Due to the exploratory nature of this set of analyses, and in the service of spurring future research, α was not adjusted.

Statistical power was sufficient for all analyses. The primary evaluation of waiver versus registry groups was powered to detect even the smallest of effects (i.e., precise Cohen’s *f* ^2^ = .019; Selya et al. 2012) based on a desired power of .80 and FDR-adjusted alpha of .029. Although exploratory, the regression analyses predicting child improvement were nonetheless sufficiently powered, as they were powered to detect small effects (i.e., precise Cohen’s *f* ^2^ = .06; Selya et al. 2012) based on a desired power of .80 and alpha of .05, and a maximum of 10 model predictors.

## Results

### Preliminary Analyses

In total, 9.73 % of values were missing, and only one noteworthy missingness pattern emerged—absence of values for time on the waiver or registry (25.4 % of participants). Missing data were handled using multiple imputation when carrying out inferential statistical tests. Auxiliary variables were included in the multiple imputation procedures and included all respective covariates, dependent variables, and independent variables. Where multiple imputation was adopted in the current study, we report pooled versions of test statistics and degrees of freedom. All degrees of freedom were adjusted based on recommendations and procedures outlined in Barnard and Rubin (1999), Enders (2010), and Reiter (2007). For the matching procedure, pooled propensity scores were derived from the multiple imputation procedure.

Descriptive statistics for the total waiver group and each matched group are presented in Table 1. Diagnostic tests confirmed that the propensity score matching procedure was effective for aligning the waiver and registry groups on family household income, child age, the number of years the child has received waivers services or been waiting on the registry, and ASD severity (see Rubin 2001; Stuart 2010, for more on diagnostic tests and evaluative criteria). Conventional diagnostic procedures were abandoned for sex, given its categorical nature, but there was a statistically non-significant relation between sex and waiver versus registry status, χ^2^(1) = .041, *p* = .839, suggesting that groups were also well matched on sex.

### Waiver Versus Registry Comparisons

We tested a multivariate general linear model with matched waiver versus registry groups as the independent variable, five covariates (i.e., time on the waiver or registry, ASD severity, child age, family income, and child sex), and six dependent variables (i.e., family quality of life and child’s improvement over the last 12 months in academic performance, independent living skills, communication skills, relationships with peers, and problematic behavior). A pooled multivariate Wald test (Rubin 1987) yielded a statistically significant model, *F*(7,261.78) = 1266.69, *p* < .0001. Specifically, multivariate tests were statistically significant for family income, *t*(253.51) = 2.87, *p* < .0042, and waiver versus registry status, *t*(271.34) = −5.08, *p* < .0001. However, ASD severity [*t*(261.67) = −2.11, *p* = .0351], child age [*t*(248.56) = −0.04, *p* = .9689], time on the waiver or registry [*t*(197.92) = 0.32, *p* = .7521], and child sex [*t*(259.85) = 0.58, *p* = .5592] were not statistically significant. Post hoc tests were performed using general linear modeling to identify relations between each dependent variable and waiver versus registry status. Results from the post hoc tests are illustrated in Table 1 and suggest that participants in the waiver group reported more improvement on independent living skills and family quality of life over the last year compared to those on the registry.

Among the dependent variables, the FQoLS total score demonstrated the strongest effect in favor of waiver services (Cohen’s *d* = 0.50). For this reason, the total score was subsequently divided into its subscales for further analyses. A single multivariate model regressed all five subscales of FQoLS on waiver versus registry status as the independent variable plus five covariates (i.e., time on the waiver or registry, ASD severity, child age, child sex, and family income). The pooled multivariate Wald test (Rubin 1987) yielded a statistically significant model, *F*(7,263.43) = 936.91 *p* < .0001. Each subscale of the FQoLS was statistically significantly higher for waiver status compared to registry status: Family Interaction, *t*(270.84) = −3.88, *p* < .0001; Parenting, *t*(268.35) = −3.79, *p* < .001; Emotional Well-being, *t*(271.99) = −5.34, *p* < .0001; Physical/Material Well-being, *t*(269.46) = −3.33, *p* < .001; and Disability-Related Support, *t*(270.84) = −4.72, *p* < .0001.

### Exploratory Analyses: Predicting Waiver Outcome

Table 2 provides the results of the regression analyses. All omnibus tests across all imputed datasets were statistically significant, except for the outcome variable of child improvement in problematic behavior, which was therefore omitted from the table. Among the service predictors, more intensive individual support services was statistically associated with increased child improvement in academic performance, independent living skills, and family quality of life over the last year. Similarly, higher frequency ratings of therapeutic integration for the child were predictive of child improvement in academic performance and family quality of life over the last year. Among the non-service predictors in the models (i.e., sex, age, years on waiver, ASD severity, and family income), the results mainly suggest a pattern in which children with more severe ASD experienced less improvement over the last year. Indeed, the rating of ASD severity was the only predictor that was statistically associated with all domains of perceived improvement (e.g., peer relationships, academic performance).

**Table 2:** Exploratory predictor analysis of participant-reported improvement: unstandardized regression weights and pooled model R^2^ from multiple regressions

Predictors	Outcome variables—child improvement				
	Academic performance	Independent living skills	Communication skills	Peer relationships	Family quality of life
Family income (thousands $)	0.013	0.019	0.020*	0.006	0.031**
ASD severity	−0.033**	−0.031**	−0.022**	−0.041**	−0.047**
Years on waiver	−0.014	−0.027**	−0.016	−0.012	0.009
Child age (years)	−0.017*	0.001	−0.018**	0.001	−0.010
Child sex	−0.028	−0.048	0.012	0.025	0.001
Environmental Accessibility	−0.048	−0.046	−0.032	−0.002	0.083
Intensive support services	0.147*	0.140*	0.087	−0.005	0.323**
Therapeutic integration	0.034*	0.014	0.013	0.005	0.040*
Family training	0.032	−0.002	−0.019	0.011	−0.012
Respite care	−0.051*	−0.007	−0.016	−0.027	0.002
Pooled model *R* ^2^	.110	.073	.089	.095	.136

Except for the model *R* ^2^ figures, all numbers in the table represent unstandardized regression coefficients (based on Type III sums of squares) for each model that regressed a single outcome variable on all 10 predictors. All inferential statistical tests were carried out with multiple imputation to address missing data. ASD = autism spectrum disorder. Pooled Model *R* ^2^ = mean *R* ^2^ across imputed datasets. Sex was coded as 0 = male and 1 = female. Environmental Accessibility was coded as 0 = did not receive services in the last 6 months and 1 = received services within the last 6 months. Intensive Support Services was coded as 0 = received fewer than an average of 3 days per week of intensive individual support services and 1 = received equal to or more than an average of 3 days per week of intensive individual support services. Child problematic behavior improvement was not included in the table because the omnibus tests across imputed datasets were statistically non-significant

*n* = 282

** *p* < .01, * *p* < .05

## Discussion

The current investigation set out to replicate and extend findings on the ASD-specific 1915(c) Medicaid Home and Community-Based Services waiver program in Maryland. Our first hypothesis was confirmed; waiver status was associated with more improvement in family quality of life over the last year compared to registry status. This replicates the results of Eskow et al. (2011) in another sample and using a more rigorous methodology. Unlike Eskow et al. (2011), waiver and registry families were statistically paired using propensity score matching on family income, child age and sex, ASD severity, and the number of years the family had been receiving waiver services or been waiting on the registry. Based on these results, which afford slightly more confidence in causal inference given the matching methodology, there is a medium-sized and positive effect of waiver services on family quality of life. Gardiner and Iarocci (2012) recently inquired about the Eskow et al. (2011) findings, as they were curious about which “services accounted for the observed differences” between the waiver and registry groups (p. 9). The current study provides some data to answer this question. The exploratory analysis of predictors of improvement in the waiver group indicated that family quality of life may be most positively influenced by increased intensive individual support services and therapeutic integration for the child. Ultimately, this is an encouraging finding, given the importance of quality of life of the child with ASD (e.g., it may be a mediator of ASD problem behavior; Garcia-Villamisar et al. 2013), as well as the negative quality of life consequences of parenting a child with ASD (e.g., Johnson et al. 2011).

Our second hypothesis was partially supported. Waiver status was associated with increased perceived child improvement over the previous year in independent living skills, but not academic performance, communication skills, peer relationships, and problematic behavior. Given the age group of the current sample—young adolescence—evidence of improvement in independent living skills is critical. This group is nearing the transition to adulthood, in which independent living is a crucial stepping stone for facilitating productivity of the individual with ASD and his or her family. It is unclear why waiver status was unrelated to child improvement in the other domains, but the age of the sample may also explain the findings. As young adolescents, children in the sample may have started the study having already developed some skills for dealing with problematic behavior, communication, dealing with peers, and academic performance, precluding measurable improvement in these domains over the last year. Indeed, as the children in the waiver group had been receiving waiver services for many years before participating in the study, these other challenges (e.g., academic performance, communication) could have already improved at an early stage in care, when the child was younger. Another non-mutually-exclusive possibility is that waiver services were designed to target independent living skills to a higher degree than these other domains of improvement, especially given the ages of the children with ASD, but data are unavailable to confirm or refute this possibility. One additional possibility is that children require different service elements (to effectively target areas like communication skills) not yet offered by the waiver, suggesting a need for expansion of support. Ultimately, future research that incorporates multi-method assessments of child outcome would help clarify further.

Based on exploratory regression analyses with the waiver group, we also predicted that more improvement over the last year would be associated with increased frequency or use of environmental accessibility adaptations, intensive individual support services, family training, therapeutic integration services, and respite care. This hypothesis was partially supported; more intensive individual support services for the child predicted more improvement in academic performance and independent living skills over the last year, and higher frequency of therapeutic integration for the child was associated with improvement in academic performance. These results highlight the promise of intensive individual support services and therapeutic integration to improve the academic outcome of children on the waiver. It is unclear why increased respite care was associated with less improvement in academic performance among those on the waiver. This finding requires further investigation, although it is possible that increased use of respite care indicates fewer hours that a family member or treatment provider is directly helping a child with his or her academic work. Although no data are available on the details of typical respite care delivery, it is unlikely that respite care providers were tasked with aiding children in the completion of academic work.

A consistent finding in the exploratory regression analysis concerned ASD severity. A higher level of severity was a negative predictor of perceived improvement over the last year in all domains (except problematic behavior, in which there was no relation). This is not surprising—severe ASD is more difficult to treat and may be more refractory to common services. Future research with bigger sample sizes would benefit from exploring ASD severity as a moderator of the relationship between frequency of service use and child improvement. Nonetheless, the findings of the current study suggest that Maryland might benefit from expanded waiver resources for dealing with the more severe segment of children with ASD. Expanded resources might facilitate the inclusion of more evidence-based techniques into the list of approved waiver services, training of more practitioners, and creation of schools and treatment settings designed to meet the specific needs of children with more severe ASD.

The current study has limitations that require acknowledgment. The cross-sectional design limits causal inference regarding the influence of waiver services on child improvement. Despite the strong diagnostics indicators supporting the success of the propensity score matching—which enhances confidence in drawing causal conclusions with non-experimental designs—it is possible that the waiver and registry families differed in some important way not specified in the propensity score model. For example, with respect to non-waiver services utilized in the last year, there were too many to include as statistical covariates and matching variables. However, univariate analyses indicate that the waiver and registry groups did not statistically differ in terms of the frequency of usage in the last year with a wide range of non-waiver services (e.g., early intervention, special education, occupational therapy, speech therapy). Nonetheless, future research would benefit from a prospective design that tracks matched waiver and registry families over time. One lurking variable that was not addressable given the data collection was group differences in cognitive functioning. Future research would benefit from matching on this individual differences variable. In addition, children on the waiver must receive a comprehensive evaluation to confirm ASD diagnosis, but children on the registry do not receive the same evaluation, suggesting that some children on the registry may not formally meet criteria for ASD. Nonetheless, to address this concern, the propensity score matching procedure successfully matched participants on ASD severity, which was rated at a high level for both the waiver and registry groups (see Table 1 for descriptive data). Last, common to community-based survey research, a large percentage of families opted not to participate in the survey. This could result in biased data, as families who volunteered and completed the survey may have specific characteristics that moderate survey responses.

There are some limitations to the survey instrument. The MASS-R contains many sections that have not been psychometrically validated, although considerable care was taken during the construction of the previous version by using focus groups of professionals and families with children with ASD. Furthermore, the patterns of MASS-R findings in the current study (direction and size of effects; adequate Cronbach’s α for the ASD severity total score) were generally consistent with expectations. Future revisions of the MASS-R instrument would also benefit from expanding the Likert scale on the items that measure child improvement to include more variability in participant responses. Another concern with the MASS-R is that measurement of improvement is entirely based on the perspective of a parent or other guardian (e.g., grandmother), which can be affected by factors like parental stress (e.g., Stokes et al. 2011). Seemingly straightforward ratings, like frequency of past service use, can be difficult to recall and report accurately by parents. Future research would benefit from evaluating this research question using more sophisticated assessment techniques, and frequency of service use could be corroborated with administrative data from treatment providers or government agencies.

Maryland maintains a comprehensive and thus expensive ASD-specific 1915(c) Medicaid Home and Community-Based Services waiver program. Eskow et al. (2011) provided preliminary evidence for the benefits of the waiver program to families, and the current study has reinforced this conclusion and extended it to provide additional evidence that the Maryland waiver might be associated with family and child improvement. Future research should focus on prospectively following waiver participants to learn whether the waiver services appear to be reaching an expressed goal of the program, which is preventing out-of-home placement.

In conclusion, while data on child outcomes post-waiver are lacking, findings from the current study are encouraging and expand the growing research base on ASD waiver programs (e.g., Timberlake et al. 2014; Warfield et al. 2013). The waiver might be associated with improvements in a child’s independent living skills and family quality of life. Other than Maryland, which maintains the largest autism waiver, nine other states have adopted a 1915(c) waiver for autism, and seven more have reported interest in doing so (Merryman et al. 2014). Thus, this promising Maryland approach could serve as a useful model for other regions who wish to adopt ASD services to meet the heavy demands of a large public health burden.

## Footnotes

1 Throughout this paper, the word *improvement* is shorthand for a more precise definition, “the perception of improvement by the participant-rater”.
